# Tumor volume features predict survival outcomes for patients diagnosed with diffuse intrinsic pontine glioma

**DOI:** 10.1093/noajnl/vdae151

**Published:** 2024-08-30

**Authors:** D’Andre Spencer, Erin R Bonner, Carlos Tor-Díez, Xinyang Liu, Kristen Bougher, Rachna Prasad, Heather Gordish-Dressman, Augustine Eze, Roger J Packer, Javad Nazarian, Marius George Linguraru, Miriam Bornhorst

**Affiliations:** Center for Genetic Medicine Research, Children’s National Hospital, Washington, District of Columbia, USA; Institute for Clinical and Translational Science, University of California, Irvine, California, USA; Center for Genetic Medicine Research, Children’s National Hospital, Washington, District of Columbia, USA; Sheikh Zayed Institute for Pediatric Surgical Innovation, Children’s National Hospital, Washington, District of Columbia, USA; Sheikh Zayed Institute for Pediatric Surgical Innovation, Children’s National Hospital, Washington, District of Columbia, USA; School of Medicine and Health Sciences, The George Washington University, Washington, District of Columbia, USA; Department of Oncology, University Children’s Hospital Zürich, Zürich, Switzerland; Department of Biostatistics, Children’s National Hospital, Washington, District of Columbia, USA; Center for Genetic Medicine Research, Children’s National Hospital, Washington, District of Columbia, USA; Brain Tumor Institute, Children’s National Hospital, Washington, District of Columbia, USA; Brain Tumor Institute, Children’s National Hospital, Washington, District of Columbia, USA; School of Medicine and Health Sciences, The George Washington University, Washington, District of Columbia, USA; Center for Genetic Medicine Research, Children’s National Hospital, Washington, District of Columbia, USA; Sheikh Zayed Institute for Pediatric Surgical Innovation, Children’s National Hospital, Washington, District of Columbia, USA; Stanley Manne Children’s Research Institute at Lurie Children’s, Chicago, Illinois, USA; Department of Hematology, Oncology, Neuro-oncology and Stem Cell Transplant, Ann & Robert H. Lurie Children’s Hospital of Chicago, Illinois, USA; Brain Tumor Institute, Children’s National Hospital, Washington, District of Columbia, USA; School of Medicine and Health Sciences, The George Washington University, Washington, District of Columbia, USA; Center for Genetic Medicine Research, Children’s National Hospital, Washington, District of Columbia, USA

**Keywords:** diffuse intrinsic pontine glioma, MRI, SVM learning, survival outcomes, tumor volume

## Abstract

**Background:**

Diffuse intrinsic pontine glioma (DIPG) is a fatal childhood central nervous system tumor. Diagnosis and monitoring of tumor response to therapy is based on magnetic resonance imaging (MRI). MRI-based analyses of tumor volume and appearance may aid in the prediction of patient overall survival (OS).

**Methods:**

Contrast-enhanced T1- and FLAIR/T2-weighted MR images were retrospectively collected from children with classical DIPG diagnosed by imaging (*n* = 43 patients). MRI features were evaluated at diagnosis (*n* = 43 patients) and post-radiation (*n* = 40 patients) to determine OS outcome predictors. Features included 3D tumor volume (T_wv_), contrast-enhancing tumor core volume (T_c_), T_c_ relative to T_wv_ (T_C_/T_wv_), and T_wv_ relative to whole brain volume. Support vector machine (SVM) learning was used to identify feature combinations that predicted OS outcome (defined as OS shorter or longer than 12 months from diagnosis).

**Results:**

Features associated with poor OS outcome included the presence of contrast-enhancing tumor at diagnosis, >15% T_c_/T_wv_ post-radiation therapy (RT), and >20% ∆Tc/T_wv_ post-RT. Consistently, SVM learning identified T_c_/T_wv_ at diagnosis (prediction accuracy of 74%) and ∆T_c_/T_wv_ at <2 months post-RT (accuracy = 75%) as primary features of poor survival.

**Conclusions:**

This study demonstrates that tumor imaging features at diagnosis and within 4 months of RT can predict differential OS outcomes in DIPG. These findings provide a framework for incorporating tumor volume-based predictive analyses into the clinical setting, with the potential for treatment customization based on tumor risk characteristics and future applications of machine-learning-based analysis.

**Key Points**

T_c_/T_wv_ ratio predicts survival outcomes in patients with diffuse intrinsic pontine glioma (DIPG).
Change in T_c_/T_wv_ ratio pre–post radiation therapy is a predictor of survival outcomes in DIPG.
T_wv_ change provides limited information about DIPG outcomes.

**Importance of the Study**

Diffuse intrinsic pontine glioma (DIPG), a diffuse midline glioma, is a fatal childhood central nervous system tumor with a median overall survival of less than one year from diagnosis. MRI is used to diagnose and monitor tumor response to therapy. However, the potential application of tumor imaging features for the prediction of patient survival outcomes remains incompletely recognized. This study utilized manual volume segmentation and machine-learning analysis of contrast-enhanced T1 and FLAIR/T2-weighted MRI images to identify imaging risk factors associated with poor survival outcomes in children diagnosed with DIPG. Our findings provide an opportunity to incorporate volumetric analysis of MRIs into DIPG management, to identify patients with variable overall survival and inform treatment strategies.

Diffuse intrinsic pontine gliomas (DIPGs), classified as diffuse midline glioma (DMGs) with or without H3K27M mutations based on WHO guidelines, are highly aggressive infiltrative tumors of the pons that are most often diagnosed in children (median age of diagnosis between 6.5 and 10 years of age) and have a median overall survival (OS) outcome of approximately 1 year from the time of diagnosis.^1–3^ Magnetic resonance imaging (MRI) is the gold standard for DIPG diagnosis and monitoring of tumor response to therapy. MRI features of DIPG have been shown to be important for diagnosis and prognosis and could be an important tool for identifying higher-risk cases early on and adapting therapy strategies accordingly for children diagnosed with DIPG and other CNS tumors.^4–8^ Diagnostic tumor MRI features, including the presence of T1 contrast enhancement within the tumor and differences in T2 appearance or texture (ie, heterogeneity or homogeneity), have shown promise for predicting patient progression-free^9^ and overall survival (OS) outcomes in children diagnosed with DIPG.^10–12^ Post-treatment MR scans have been evaluated to further characterize tumor behavior.^13^ MRI data including metabolic ratios, perfusion metrics, and signal intensity has been evaluated for associations with OS and treatment response.^14–18^ More recent studies have proposed risk stratifications for DMG based on MRI features,^18,19^ highlighting the future potential of MRIs as a less invasive means of classifying patients with newly diagnosed DMG. Despite these recent studies, the predictive value of analyzing MR images obtained at upfront diagnosis and longitudinally post-treatment for children diagnosed with DIPG remains incompletely defined.

Studies have shown that the evaluation of tumor 3D volumetric measurements provides a more accurate quantification of tumor size when compared to 2D cross-products.^20^ For example, changes in whole tumor volume following standard-of-care radiation therapy (RT) can be indicative of tumor response to treatment and used to predict the onset of disease progression in children with DIPG.^21^ Similarly, in adult glioblastoma (GBM), baseline and post-treatment enhancing tumor volume, percent change in enhancing tumor volume, and post-treatment whole tumor volume, have been associated with survival outcomes.^22^

More recently, machine learning has demonstrated utility in MR image volumetric analysis and subsequent characterization of tumor features.^23–26^ Different machine learning algorithms have been proposed to use certain MR features for tumor volume segmentation,^27,28^ monitoring treatment progression,^29^ and predicting clinical outcomes.^30,31^ In adult patients with GBM, subdividing tumor volumes into different regions (eg, based on high versus low cellularity) improved machine learning-based prediction of survival outcomes for recurrent GBM, highlighting the prognostic value of quantifying tumor imaging features including heterogeneity.^22,32^ Machine learning algorithms for adult brain tumors have also been analyzed for their transferability to the pediatric population, increasing the application of these algorithms for patients with DMGs.^33–35^

Utilization of multi-pronged approaches can provide practitioners with a more comprehensive set of tools to provide the most optimal care for patients with DIPG. Both manual and machine-based segmentation techniques have shown the potential to identify tumor characteristics of low- and high-risk patients, enabling more precise care, which may lead to prolonged survival outcomes. In this study, contrast-enhanced T1 and FLAIR/T2-weighted MR images were obtained from children and young adults diagnosed with DIPG. Utilizing these images, this study aims to identify tumor volumetric features that may be predictive of survival outcomes in pediatric populations.

## Materials and Methods

### Collection of Patient Clinical and Imaging Data

Prior to data collection, patients either consented to the Children’s National Hospital Institutional Review Board-approved study Pro00001339 (PI Dr. Nazarian) or the exempt protocol Pro00003792 (PI Dr. Linguraru). Pediatric and adolescent patients (*n* = 46) treated for DIPG between 2010 and 2019 at Children’s National Hospital were identified retrospectively for the study. All patients had a classic (“typical”) DIPG based on radiological imaging defined as T1-hypointense and T2-hyperintense diffusely infiltrative tumors that arise from the pons and involve at least 50% of the pons by cross-sectional area.^36^ Of all the patients,56% (*n* = 24), underwent biopsy at the time of diagnosis. Three patients were excluded from analysis for insufficient clinical/survival or imaging data (ie, no contrast given, poor imaging quality, no clinical data available). Contrast-enhanced T1 and FLAIR/T2-weighted MR images were obtained at diagnosis from the remaining (*n* = 43) pediatric and adolescent patients (Table 1, Supplementary Table 1). Additionally, images were obtained at the first available timepoint following initial standard of care RT between 1 day and 2 months post-RT (<2 mos post-RT; *n* = 37 patients with imaging data available at this time point), and at a second follow-up timepoint between 2 to 4 months post-RT (2–4 mos post-RT; *n* = 37 patients; Supplementary Table 1). One patient passed away during RT, and 2 additional patients did not have a post-RT MRI prior to 6 months after treatment, and thus were excluded from the post-RT analysis. All other patients (*n* = 40) had at least one post-RT MRI.

**Table 1.** Demographics and Clinical Characteristics of Diffuse Intrinsic Pontine Glioma Patients Included in the Analysis

Patient cohort (*n* = 43)	
Age (years)	
Median	6.1
Range	3.2–25.9
Gender	
Male	19 (44%)
Female	24 (55%)
H3K27 status	
H3.1K27M	3 (7%)
H3.3K27M	20 (47%)
H3WT	1 (2%)
N/A	19 (44%)
TP53 status	
Mutant	12 (28%)
WT	3 (7%)
N/A	28 (65%)
OS (months)	
Median	11.7
Range	3.3–43.1
Radiation treatment	
Diagnosis	43 (100%)
Repeat RT	10 (23%)
Additional treatment received	
Chemotherapy	12 (28%)
Molecular or epigenetic ± chemotherapy	18 (42%)
Immunotherapy ± molecular or chemotherapy	8 (19%)
Avastin	2 (4%)
RT only	3 (7%)

## Tumor Volume Segmentation

**Figure 1.**
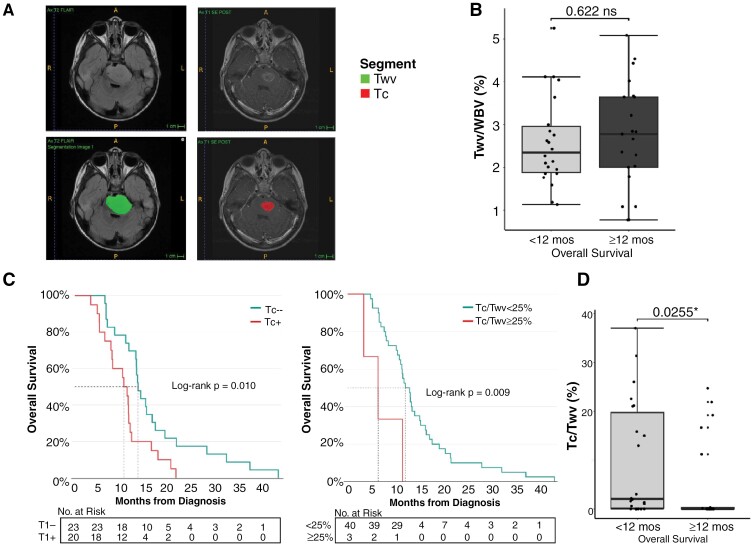
Diagnostic tumor MRI volume is predictive of survival outcomes (A) Representative axial MRI from one patient showing overlaying T_wv_ and T_c_ tumor volume segmentations. (B) T_wv_ relative to whole brain volume (WBV) between shorter (*n* = 22) versus longer (*n* = 21 with available WBV data) survivors. Mann–Whitney test, *P* = .622. (C) *Left:* Survival comparison of patients with contrast-enhancing tumor on T1 imaging (T_c_+) (*n* = 23) versus those without contrast-enhancing tumor on T1 imaging (T_c_−) (*n* = 20), Log-rank test, *P* = .010. *Right:* Survival comparison of patients with T_c_/T_wv_ > 25% (*n* = 3) vs. those with T_c_/T_wv_ < 25% (*n* = 40) at diagnosis, Log-rank test, *P* = .009. (D) Comparison of T_c_/T_wv_ (%) between shorter (OS < 12 months, *n* = 22) versus longer survivors (OS ≥ 12 months, *n* = 21), Mann–Whitney test, *P* = .0255.

The open-source ITK-SNAP software (itksnap.org^37^) was used to segment and obtain measurements of total tumor volume using 2D FLAIR/T2-weighted images and of contrast-enhancing tissue using 2D contrast-enhanced T1-weighted images. Since FLAIR images were obtained most frequently, these were preferred for analysis of tumor volume (*n* = 49 FLAIR, *n* = 48 FLAIR plus gadolinium). In cases where FLAIR images were not available, other types of T2-weighted images were used including T2-weighted fast-spin echo (T2FSE) (n = 6 images), T2-weighted propeller (*n* = 3), and T2-weighted (*n* = 11) sequences. T1 post-gadolinium images were used for T_c_ segmentation. Segmentations were generated using axial plane images (Figure 1A). Slice thickness was between 3 and 5 mm for all images. All T_wv_ and T_c_ segmentations were conducted by trained laboratory personnel (ERB and KB) and were reviewed by a neuro-oncologist (MB). A representative subset of segmentations were then jointly reviewed by a Neuro-radiologist (GV) and neuro-oncologist (MB) to ensure the adequacy of the segmentation technique and the accuracy of the data. Following segmentation, the proportion of T_c_ relative to T_wv_ volume (T_C_/T_wv_) was calculated. To account for nonspecific artifacts in the MRIs, patients were defined as T_c_ “positive” (‘T_c_+’) if T_c_/T_wv_ was greater than 1% (ie, T_c_ volume comprised more than 1% of total tumor volume). Total T_wv_ volume percent change from baseline was calculated as: (T_wv post-RT—_T_wv baseline_)/T_wv baseline_ x 100%.

### Machine Learning Classification

Tumor features were extracted as defined by tumor segmentations. Two different analyses were performed, the first analysis including data from diagnostic MRIs, and a second analysis including single timepoint and comparison features between the diagnosis and <2 months post-RT. Features that were calculated at each timepoint are described below:

1. Volumetric features diagnosis only (8): T_c_/T_wv_ seg ratio, T_c_ seg absolute volume, T_c_ seg volume relative to whole brain volume (WBV), difference of T_wv_ to T_c_ seg volume relative to WBV, difference of T_wv_ to T_c_ seg volume, T_wv_ seg volume relative to WBV, WBV, T_wv_ seg absolute volume.
2. Volumetric features diagnosis and <2 months post-RT (24): T_c_/T_wv_ ratio change (diagnosis to <2 months post-RT), T_c_/Twv ratio (<2 months post-RT), T_c_ seg absolute volume (<2 months post-RT), T_c_ seg volume relative to WBV (<2 months post-RT), T_c_ seg volume relative to WBV change (diagnosis to <2 months post RT), T_c_ seg volume change (<2 months post-RT to diagnosis), T_c_ seg absolute volume (diagnosis), T_wv_ seg absolute volume relative to WBV change (<2 months post-RT to diagnosis), T_c_/T_wv_ ratio (diagnosis), T_wv_ seg absolute volume relative to WBV (<2 months post-RT to diagnosis), T_c_ seg absolute volume relative to WBV (diagnosis), T_wv_ seg absolute volume (<2 months post-RT), difference of T_wv_ and T_c_ seg volume relative to WBV change (diagnosis to <2 months post-RT), T_wv_ seg absolute volume change (diagnosis to <2 months post-RT), difference of T_wv_ and T_c_ seg absolute volume (<2 months post-RT), difference of T_wv_ and T_c_ seg volume relative to WBV (<2 months post-RT), difference of T_wv_ and T_c_ seg absolute volume change (diagnosis to <2 months post-RT), WBV (diagnosis), WBV (<2 months post-RT), WBV change (diagnosis to <2 months post-RT), T2 seg volume relative to WBV (diagnosis), difference of T_wv_ and T_c_ seg volume relative to WBV (diagnosis), T_wv_ seg absolute volume (diagnosis), difference of T_wv_ and T_c_ seg absolute volume (diagnosis).
3. Included demographic features at both timepoints (2): patient age and gender.

To calculate whole brain volume (WBV), images were preprocessed with an automated tool, FeTS (https://fets-ai.github.io/Front-End/). The preprocessing includes rigid registration of T1 and T2 images to the SRI-24 Atlas^38^ and a skull-stripping,^39^ which separates brain tissue from non-brain tissue. The whole brain volume was then calculated using PyRadiomics (https://pyradiomics.readthedocs.io/en/latest/#) based on the skull-stripped brain mask.

After the above features were calculated, they were standardized by removing the mean and scaling to unit variance. Feature ranking with recursive feature elimination (scikit-learn.org) was performed with a linear support vector machine (SVM) estimator to select the most *n* important features for OS classification. To avoid overfitting the model to the training data, the *n* was capped to be <10% of the number of patients. Classification results using the *n* selected features and the same SVM classifier were reported.

### Statistical Analyses

Mann–Whitney tests were used to compare imaging features (eg, T_c_ volume) between OS groups (shorter vs. longer than 12 months survival). Log-rank (Mantel-Cox) tests were used to compare OS outcomes between comparison groups. Wilcoxon signed-rank test with Bonferroni multiple testing correction was used to compare T_c_/T_wv_ values between pre- and post-RT at <2 and 2–4 months post-RT (paired comparisons). Time-dependent univariable cox proportional hazards were calculated utilizing variables (continuous and categorical) associated with SVM classification to assess the relationship of factors related to progression-free survival (PFS), or tumor progression (defined by T_wv_ change of ≥25% as per RANO criteria^40,41^). Variables were only included if they had <10% missing data and outcomes with greater than 10 events. Statistical analyses and plot generation were performed using R Studio (Rstudio Team, 2020). Statistical tests were reviewed with a biostatistician (HGD).

## Results

### Effect of Patient Demographics and Therapy on Overall Survival

In total, 43 children and young adults diagnosed with DIPG were included in the study (male, 44%, *n* = 19; female, 56%, *n* = 24, Table 1). Subjects were diagnosed at a median age of 6.1 years and experienced a median overall survival (OS) time of 11.7 months from diagnosis (range 3.3–43.1 months), consistent with the typical outcome for this patient population.^1,4,42^ All patients who underwent a tumor biopsy had H3K27M mutation testing performed (*n* = 24), and a majority also had TP53 mutation testing (*n* = 15). Of these patients, 96% (*n* = 23/24) harbored histone H3K27M mutation (H3.1K27M, *n* = 3; H3.3K27M, *n* = 20; H3.1/H3.3WT, *n* = 1) and 80% (*n* = 12/15) harbored mutant *TP53*, consistent with previous publications^1,43^ (Table 1).

All patients received at least a single course of standard-of-care RT, with 23% (*n* = 10) of patients receiving 2 or more courses of RT (Table 1). Most patients were enrolled in or treated as per a clinical trial (88%, *n* = 38/43), with the primary treatment agent being categorized as molecular-targeted therapy (42%), chemotherapy (28%), or immunotherapy (19%; Table 1). Three patients received RT alone (*n* = 1 repeat RT) and 2 patients received RT with Avastin (*n* = 1 repeat RT). Most patients were not on therapy (26/37, 70%) at the time of the first MRI (Supplementary Table 1). In contrast, by the second MRI, the majority of patients were on therapy (28/37, 76%; Supplementary Table 1).

Patient age at diagnosis and sex did not influence OS outcomes (Supplementary Figure 1A–1C). Subjects that underwent repeat RT experienced longer median OS compared to those that received a single course of radiation (*P* = .029; Supplementary Figure 1D), though this may be due to increased eligibility for repeat RT in patients who already have had longer survival. Treatment type did not affect OS outcomes among the cohort (*P* > .9, Supplementary Figure 1E).

While previous studies have shown a difference in OS outcome based on H3K27 and *TP53* mutation status,^1,42,44^ among this cohort there was no significant impact of mutation status on survival. However, many of the patients included in this study were diagnosed before biopsy/tissue analysis became a common practice, particularly for clinical trial enrollment, which limited this analysis as many patients did not have molecular data available (44% with unknown H3K27 status and 65% with unknown TP53 status).

### The Presence and Proportion of T1 Contrast-Enhancing Tumor at Diagnosis Predicts Survival Outcome

Patients were stratified into “short survivors” (OS < 12 months from diagnosis, *n* = 22) and “long survivors” (OS ≥ 12 months from diagnosis, *n* = 21), based on this study cohort’s median OS of 11.7 months, which is consistent with the published median survival for patients with DIPG in literature.^1,2^ T_c_ and T_wv_ tumor volumes were calculated using manual segmentation for all patients at diagnosis (Figure 1A). We first calculated total tumor volume relative to whole brain volume as a single feature using T_wv_ images and found no difference between the short and long survivors (Figure 1B). In contrast, the presence of a contrast-enhancing tumor (Tc+) at diagnosis predicted a significantly shorter OS outcome (Log-rank Mantel-Cox test, *P* = .010, Figure 1C, *left*). Consistently, short survivors exhibit a significantly higher T_c_/T_wv_ ratio (T_c_/T_wv_ (%)) at diagnosis (Mann–Whitney test, *P* = .0255, Figure 1D). This difference was not influenced by patient age or gender (Supplementary Figure 2A and 2B). We explored different thresholds of T_c_/T_wv_ ratio that could be most predictive of survival outcomes and found that patients with ≥25% T_c_/T_wv_ (*n* = 3) had significantly lower OS than patients with T_c_/T_wv_ < 25% (*n* = 40) (median survival 6 vs 11 months; *P* = .009; Figure 1C, right).

Collectively, diagnostic MRI analyses showed that the presence of contrast-enhancing tumors on T1 images and a high proportion (>25%) of T_c_ relative to T_wv_ volume independently predicted poor OS outcomes in children diagnosed with DIPG (Figure 3F).

### Increase in T1-Contrast-Enhancing Tumor Following RT Informs OS Outcome

**Figure 2.**
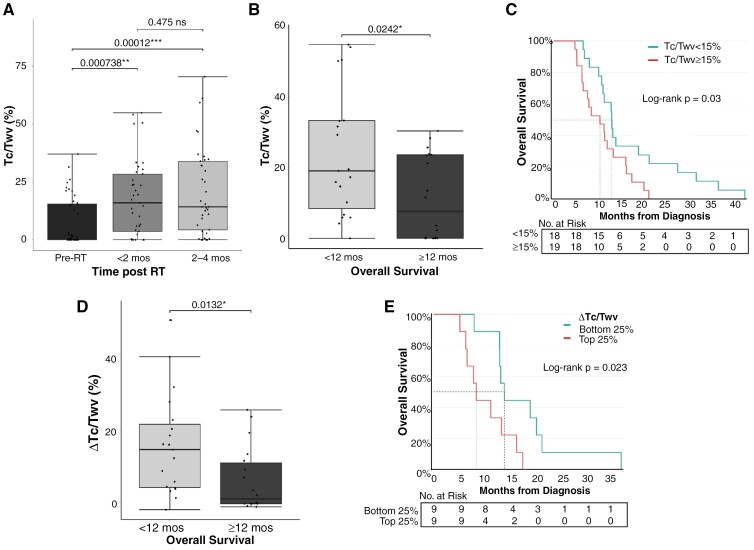
T_c_/T_wv_ following radiation therapy predicts survival outcomes. (A) Comparison of T_c_/T_wv_ at initial diagnosis (‘Pre-RT’; *n* = 43) and following RT at <2 months (*n* = 37; Wilcoxon signed rank, corrected *P* = .0007) and 2–4 months post-RT (*n* = 37; Wilcoxon signed rank, corrected *P* = .0001). There was no significant difference in the T_c_/T_wv_ ratios between the <2 months and 2–4 months MRI timepoints (*P* = .475) (B) Difference in T_c_/T_wv_ at < 2 mo post-RT between shorter (*n* = 19) and longer (*n* = 18) survivors, Mann–Whitney test, *P* = .0242. (C) Survival comparison between patients with ≥15% T_c_/T_wv_ (*n* = 19) and those with <15% T_c_/T_wv_ (*n* = 18) at <2 months post-RT, Log-rank test, *P* = .03. (D) Difference in ∆T_c_/T_wv_ from diagnosis to <2 mo post-RT between shorter (*n* = 19) and longer (*n* = 18) survivors, Mann–Whitney test, *P* = .0132. (E) Survival comparison at the <2 months post-RT timepoint between patients with a large ∆T_c_/T_wv_ increase (*n* = 9; ∆ T_c_/T_wv_ > 19%, top 25% of all patients) and patients within the bottom 25% (*n* = 9; ∆ T_c_/T_wv_ between −1.6% and 0.15%), Log-rank test, *P* = .023.

MRIs collected following the completion of RT were then evaluated to identify post-treatment imaging features predictive of OS outcome. The initial focus was on images obtained at an early timepoint following completion of RT, within 1 day to 2 months post-treatment (*n* = 37 patients with available <2 mos post-RT imaging data). This timepoint was prioritized given the clinical relevance of identifying early predictors of OS outcome, and the lower number of patients receiving treatment that could potentially influence the results. The Tc/T_wv_ ratio significantly increased from the diagnostic to the <2 months post-RT MR image, suggesting increased contrast uptake in the tumor in response to RT (Wilcoxon signed rank test, corrected *P* = .0007; Figure 2A). In the second follow-up MRI scan obtained 2–4 months post-RT, the mean T_c_/T_wv_ ratio was not significantly different than the initial follow-up MRI (*P* = .475), but still significantly higher than at diagnosis (Wilcoxon signed rank test, corrected *P* = .0001; Figure 2A).

At <2 months post-RT, the binary presence–absence of T_c_ no longer predicted OS outcome (Supplementary Figure 3A) in contrast to the diagnostic MRI (Figure 1C; left). However, the T_c_/T_wv_ ratio significantly differed between short and long survivor groups (Figure 2B), with short survivors exhibiting higher T_c_/T_wv_ ratios (Mann–Whitney test, *P* = .0242). Similar to the analysis at diagnosis, T_c_/T_wv_ thresholds were explored and patients with ≥15% T_c_/T_wv_ exhibited a shorter OS than patients with <15% Tc/T_wv_ post-RT (*P* = .03, Figure 2C). Comparing different T_c_/T_wv_ thresholds revealed that OS outcomes were most significantly different as the threshold increased, with the most significant threshold of T_c_/T_wv_ seen at 30% (*P* = .004, Supplementary Figure 3B).

**Figure 3.**
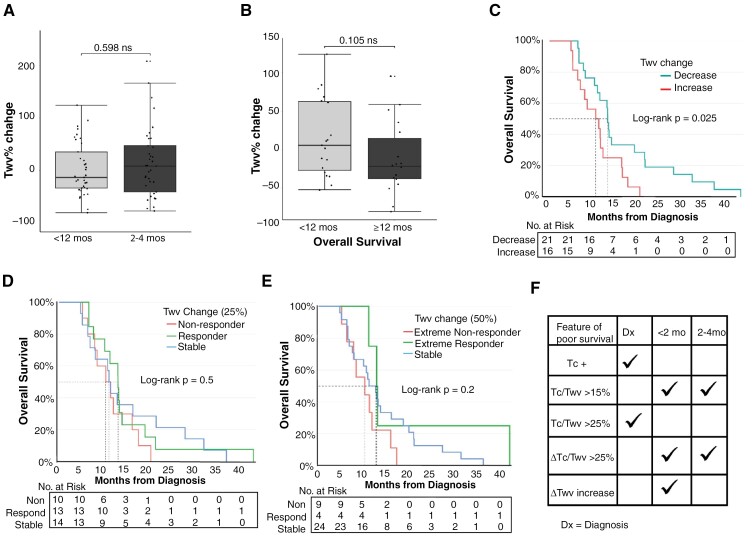
Whole tumor volume changes post-radiation provide limited insight into patient survival outcomes. (A) Change in T_wv_ from baseline to <2 months RT and baseline to 2–4 months post-RT (*n* = 37 patients for each timepoint). There was no difference in T_wv_ volume change between the 2 timepoints (Wilcoxon matched-pairs signed rank test, *P* = .598). (B) T_wv_ percent change at <2 months post-RT between shorter and longer survivors. Mann–Whitney test, *P* = .105. (C) Survival comparison between patients who experienced a decrease (*n* = 21) versus an increase (*n* = 16) in T_wv_ volume from diagnosis to <2 months post-RT, Log-rank test, *P* = .025. (D) OS outcome of patients who experienced a T_wv_ percent increase greater than 25% of the initial diagnosis volume (“non-responders”) (*n* = 10), decrease greater than 25% (‘responders’) (*n* = 13), or a T_wv_ percent change less than 25% (“stable”) (*n* = 14). Log-rank test, *P* = .5. (E) OS outcome of patients who experienced a T_wv_ percent increase greater than 50% of the initial diagnosis volume (extreme “non-responders”) (*n* = 9), decrease greater than 50% (extreme ‘responders’) (*n* = 4), or a T_wv_ percent change less than 50% (“stable”)(*n* = 24). Log-rank test, *P* = .2. (F) Summary of MRI volume features at each timepoint that are independently predictive of OS.

Given the observed large increase in T_c_/T_wv_ between diagnostic and post-RT MRIs (Figure 2A), survival implications of ∆T_c_/T_wv_ following radiotherapy were considered. Shorter survivors had a significantly larger ∆T_c_/T_wv_ (increase) when compared to longer survivors (Mann–Whitney test, *P* = .0132, Figure 2D). Patients who experienced the largest increase in T_c_/T_wv_ from baseline to the first follow-up scan (∆T_c_/T_wv_ in the top 25% of all patients, range 19.2% to 49.9%; median 25% change) had a significantly shorter OS when compared to those with the smallest change (∆ T_c_/T_wv_ in the bottom 25%, range −1.6% to 0.2%; median 0% change; *P* = .023; Figure 2E). In summary, the most significant predictors of poor survival based on T_c_/T_wv_ ratio at <2 months and 2–4 months after RT include T_c_/T_wv_ ratio >15% and change in T_c_/T_wv_ ratio after RT >25% (Figure 3F).

### Total Tumor Volume (T_wv_) Provides Limited Predictive Insight into OS Outcome

When considering T_wv_ volume percent change from baseline, a broad range of volumetric changes were observed, ranging from extremes of −81% to +124% of the baseline diagnostic T_wv_ volume at <2 months post-RT (median = −13.4%) and −77% to +208% of the baseline diagnostic T_wv_ volume at 2–4 months post-RT (median = 8%; Figure 3A). There was no significant difference in T_wv_ volume change between the <2 months and 2–4 months post-RT timepoints (*P* = .598, Figure 3A). When comparing T_wv_ percent change between shorter and longer survivors, there was a trend toward increased T_wv_ change at <2 months post-RT in shorter survivors, but this was not significant (*P* = .105, Figure 3B). Patients with any level of increase in T_wv_ volume after RT prior to 2 months; however, had a worse survival outcome when compared to those with a decrease (*P* = .025, Figure 3C). This finding was no longer present at the 2 to 4-month post-RT timepoint (*P* = .2).

To better understand the clinical implications of this wide range of T_wv_ volumetric changes, patients were stratified into 3 groups based on <2 months post-RT imaging results: non-responders, responders, and stable patients, using 25% as the cutoff based on published RANO criteria.^40^ These groups were defined respectively as those experiencing: A volumetric increase >25% of the initial T_wv_ volume, a T_wv_ decrease >25% from diagnosis, and a T_wv_ (increase or decrease) change between 0% and 25%. There was no significant OS difference between these 3 groups, but responders showed a slightly longer median OS (13.05 months) when compared to non-responders (10.88 months) and stable (11.19 months) patients (Figure 3D). Moreover, when grouping patients into ‘extreme’ responders and nonresponders, defined as those with a decrease (responders) or an increase (nonresponders) of >50% of the baseline T_wv_ volume, there was still no significant difference between groups (Figure 3E). However, despite no difference in median OS, most of the extreme nonresponders had a survival of <12 months, and none had an overall survival greater than 18 months, suggesting that these patients have a shorter lifespan following diagnosis.

As shown, patients with T_c_/T_wv_ >15% exhibited a shorter OS than patients with <15% Tc/T_wv_ post-RT. Consistent with the above T_c_ and T_wv_ data, while there was an average increase in T_c_ values of 220% between baseline and the <2 months follow-up scan, the T_wv_ values had an average change of −2.872%. This suggests that the increased T_c_/T_wv_ at the post-RT MRI is primarily related to changes in T_c_ rather than T_wv_. Collectively, these findings reveal that while patients with T_wv_ increase from baseline to <2 months had worse overall survival (Figure 3C and 3F), changes alone provide only limited insight into tumor response and OS outcomes in children diagnosed with DIPG.

### Machine Learning Identifies Combinations of Diagnostic and Longitudinal MRI Features Predictive of Survival Outcome

Tumor volume and demographic features were combined (*n* = 10 for diagnosis alone, and *n* = 26 for diagnosis and <2 mos post-RT, see Methods), and SVM learning was applied to identify feature combinations most predictive of OS outcome (OS < 12 months vs. OS ≥ 12 months). Characteristics of the features used for SVM learning can be found in Supplementary File 1. When using only diagnostic features (this applied to 43 patients), SVM learning identified one feature, T_c_/T_wv_ ratio at diagnosis, as the most significant predictor of OS outcome (Table 2). Classification using this feature resulted in overall survival prediction accuracy of 74% (sensitivity = 73%, specificity = 76%). When combining both diagnostic and post-RT images (this applied to 36 patients), SVM learning also identified one feature, ∆T_c_/T_wv_ from diagnosis to <2 months post-RT, as the most significant predictor of OS outcome (Table 2). Classification using this feature resulted in overall survival prediction accuracy of 75% (sensitivity = 84%, specificity = 65%). Our experiments suggested using more features would not improve classification performance for the above 2 scenarios.

**Table 2.** Imaging Features Based on Support Vector Machine That Predict Patient Overall Survival Outcomes at Diagnosis and Between Diagnosis to <2 Months Post-Radiation Therapy

Feature	OS < 12 months (mean ± SD)	OS ≥ 12 months (mean ± SD)	*P*-value
T_c_/T_wv_ (Diagnosis)	0.10 ± 0.12	0.04 ± 0.08	.026
∆T_c_/T_wv_ (Diagnosis—<2 months)	0.16 ± 0.14	0.06 ± 0.09	.011

Abbreviations: OS = overall survival.

Taking SVM features that were determined to be important for survival, a time-dependent cox-proportional hazard analysis was performed to show the relationship of these variables to PFS, to identify patients who are at greatest risk for tumor progression early in their disease course. For this analysis, PFS was defined as the absence of T_wv_ increases greater than 25% at the 2 MRI follow-up timepoints (<2 mos and 2–4 mos post-RT). T_c_/T_wv_ ratio greater than 15% and an increase in T_c_/T_wv_ ratio after RT (ΔT_c_/T_wv_ ratio) were both associated with risk for early progression (shorter PFS; Table 3). Age, gender, study type, and binary presence of contrast enhancement alone were not shown to be associated with PFS (Table 3).

**Table 3.** Time-Dependent Univariable Cox Proportional Hazards Based on SVM-Related Features and Progression-Free Survival

Characteristics	*N*	HR	95% CI	*P*-value	*q*-Value^a^
Gender	92				
Female		1.00	—		
Male		1.19	0.52, 2.73	.68	0.69
Age years (continuous)	92	0.94	0.85, 1.04	.23	0.32
T_wv_	92	1.00	1.00, 1.00	.69	0.69
T_c_	92	1.00	1.00, 1.00	.051	0.12
T_c_ status	92				
T_c−_		1.00	—		
T_c_+		1.83	0.75, 4.48	.19	0.32
ΔT_c_/T_wv_	92	25.9	1.43, 469	.028	0.12
T_c_ (15%) enhancing	92				
T_c_ ≥ 15		1.00	—		
T_c_ < 15		0.41	0.18, 0.95	.036	0.12

Abbreviations: SVM = support vector machine

^a^ False discovery rate correction for multiple testing.

## Discussion

This study revealed radiographic volume features that significantly predicted patient OS outcomes in children and young adults diagnosed with DIPG. These features primarily focused on the ratio of contrast-enhancing tumor (T_c_) compared to the total tumor volume (T_wv_) at individual timepoints, and in response to RT. At diagnosis, the presence of T1 enhancing tissue and T_c_/T_wv_ ratio >25% are independently predictive of shorter overall survival (<12 months; Figures 1C and 3F). After completion of RT, T_c_/T_wv_ratio >15% and ∆T_c_/Twv >25% were predictive of shorter overall survival at both the <2 months and 2–4 months post-RT timepoints (Figures 2C and E and 3F). Change in the tumor volume (∆T_wv_) had prective value only if increased within 2 months of completion of RT (Figure 3C and F). Combined, these findings demonstrate that T_c_/T_wv_ ratio and ∆ T_c_/T_wv_ in response to RT as independent features likely have the best predictive value for short survival in patients with DIPG, a finding that was confirmed through SVM learning. This is consistent with previous studies that have also shown the presence of T_c_-enhancing tissue in DIPGs is associated with poor progression-free and overall survival.^9,45^ However, by quantitating the T_c_/T_wv_ ratio at different clinical timepoints that are most associated with OS, and by determining the change in T_c_/T_wv_ from initial diagnosis to post-RT (∆ T_c_/T_wv_), this study provides more sensitive metrics for understanding thresholds of T_c_/T_wv_ ratio associated with poor survival outcomes, allowing for better clinical translation.

While the results of this study provide a foundation for using volume changes in T_c_ and T_wv_ at diagnosis and in response to therapy as an indicative feature for outcomes, further investigation into the predictive value of the tumor characteristics and trajectory, and incorporation of these features into tumor response criteria is warranted. In adults, and many pediatric trials, response to therapy is commonly defined by the RANO (Response Assessment in Neuro-Oncology) criteria for low- and high-grade gliomas.^40,41^ This incorporates changes in contrast enhancement and FLAIR/T2 2D volume when determining tumor response, with a cutoff of ≥25% increase or ≥50% decrease in product of perpendicular diameter defining progression and response, respectively. Our findings show that a ratio of T1-contrast-enhancing tissue to FLAIR/T2 tumor volume has the best prediction of overall survival in patients. As volumetric analysis of tumors becomes more common, it is possible that single measurements may not be the best measure of tumor response to therapy, and other features or ratios could be considered in response criteria. In addition, as more volume segmentation studies are performed looking at the response to therapy, the tumor volume cutoffs may need to be altered as 3D measurements can provide a more sensitive measure of response.^46^

In adult GBM, machine learning has been shown to predict major mutation subtypes including IDH mutation^47^ and 1p/19q co-deletion status^48^ using radiographic imaging features. Imaging features, including radiographic response to RT, may similarly correlate to molecular features, as seen in a recent publication that used H3K27M mutation subtype and transcriptomic profiles along with radiographic features post-RT to identify subgroups of DIPG.^49^ Associations between tumor mutation status (H3K27M, *TP53* mutations, see Table 1) and tumor MRI volumetric features, with a particular focus on tumors with T1 enhancement, were not found in this study. However, this is likely due to the lack of available molecular data for many patients included in this retrospective study. Given this limitation, future prospective studies are warranted to dissect the contribution of tumor genomic alterations to radiographic signatures in children with DIPG.

There are several limitations to this study. Since this was a retrospective study, only images that were available could be included in the analysis, and therefore the MRI images were not the same across all timepoints for all patients. We attempted to use the same MRI technique for each patient for comparison, but there were some patients where this was not possible (Supplementary Table 1). However, since these patients were the minority, we do not feel that this significantly impacted the results of our analysis. This study included 43 patients and 117 MRIs over 3 timepoints, which is a significant number given the rare diagnosis of DIPGs but could be expanded. Although shown to not be significant based on treatment type, the impact of non-RT associated therapy on T_wv_ and T_c_ was also not well defined in this study given the small numbers of patients and a large variety of treatment types. Since this study focused on radiographic features alone, without biological correlative tissue analysis, the relationship between biological changes and increased T_c_ change remains unknown. Finally, this study focused on independent risk factors, and therefore the combined effect of multiple risk factors on outcomes is not known. A future prospective study using consistent imaging techniques and a larger number of patients who are all receiving the same treatment would be helpful to confirm the findings in this study.

The results of this study provide a foundation for using volume changes in T_c_ relative to T_wv_ at diagnosis and in response to therapy as a predictive feature for outcomes in children with DIPG. Although enhanced through machine learning and automated segmentation, the T_c_/T_wv_ volumetric analysis can be performed in nearly all environments using manual segmentation. Therefore, this type of analysis would facilitate the incorporation of tumor volumetric properties into prognostic stratification and response monitoring for children diagnosed with DIPG in multiple clinical settings.

## Supplementary Material

vdae151_suppl_Supplementary_Figues

vdae151_suppl_Supplementary_Table

vdae151_suppl_Supplementary_File_S1

## Data Availability

All imaging segmentation data is available through reasonable request.

## References

[1] Mackay A , Burford A, Carvalho D, et al. Integrated molecular meta-analysis of 1,000 pediatric high-grade and diffuse intrinsic pontine glioma. Cancer Cell. 2017;32(4):520–537.e5. 28966033 10.1016/j.ccell.2017.08.017 PMC5637314

[2] Hoffman LM , DeWire M, Ryall S, et al. Spatial genomic heterogeneity in diffuse intrinsic pontine and midline high-grade glioma: implications for diagnostic biopsy and targeted therapeutics. Acta Neuropathol Commun. 2016;4:1. 26727948 10.1186/s40478-015-0269-0 PMC4700584

[3] Louis DN , Perry A, Wesseling P, et al. The 2021 WHO classification of tumors of the central nervous system: a summary. Neuro-Oncology. 2021;23(8):1231–1251. 34185076 10.1093/neuonc/noab106 PMC8328013

[4] Hoffman LM , Veldhuijzen van Zanten SEM, Colditz N, et al. Clinical, radiologic, pathologic, and molecular characteristics of long-term survivors of diffuse intrinsic pontine glioma (DIPG): a collaborative report from the International and European Society for Pediatric Oncology DIPG Registries. J Clin Oncol. 2018;36(19):1963–1972. 29746225 10.1200/JCO.2017.75.9308 PMC6075859

[5] Giagnacovo M , Antonelli M, Biassoni V, et al. Retrospective analysis on the consistency of MRI features with histological and molecular markers in diffuse intrinsic pontine glioma (DIPG). Child’s Nerv Sys*t.* 2020;36(4):697–704. 31848724 10.1007/s00381-019-04463-y

[6] Calmon R , Dangouloff-Ros V, Varlet P, et al. Radiogenomics of diffuse intrinsic pontine gliomas (DIPGs): correlation of histological and biological characteristics with multimodal MRI features. Eur Radiol. 2021;31(12):8913–8924. 34003354 10.1007/s00330-021-07991-x

[7] Lazow MA , Fuller C, DeWire M, et al. Accuracy of central neuro-imaging review of DIPG compared with histopathology in the International DIPG Registry. Neuro-Oncology. 2022;24(5):821–833. 34668975 10.1093/neuonc/noab245 PMC9071293

[8] Steffen-Smith EA , Baker EH, Venzon D, et al. Measurements of the pons as a biomarker of progression for pediatric DIPG. J Neurooncol. 2014;116(1):127–133. 24113877 10.1007/s11060-013-1266-4 PMC6301003

[9] Wagner MW , Namdar K, Napoleone M, et al. Radiomic features based on MRI predict progression-free survival in pediatric diffuse midline glioma/diffuse intrinsic pontine glioma. Can Assoc Radiol J. 2023;74(1):119–126. 35768942 10.1177/08465371221109921

[10] Tam LT , Yeom KW, Wright JN, et al. MRI-based radiomics for prognosis of pediatric diffuse intrinsic pontine glioma: an international study. Neurooncol Adv. 2021;3(1):vdab042. 33977272 10.1093/noajnl/vdab042 PMC8095337

[11] Leach JL , Roebker J, Schafer A, et al. MR imaging features of diffuse intrinsic pontine glioma and relationship to overall survival: report from the International DIPG Registry. Neuro-Oncology. 2020;22(11):1647–1657. 32506137 10.1093/neuonc/noaa140 PMC7690352

[12] Szychot E , Youssef A, Ganeshan B, et al. Predicting outcome in childhood diffuse midline gliomas using magnetic resonance imaging based texture analysis. J Neuroradiol. 2021;48(4):243–247. 32184119 10.1016/j.neurad.2020.02.005

[13] Ko C , Kaushal A, Hammoud DA, et al. Role of early postradiation magnetic resonance imaging scans in children with diffuse intrinsic pontine glioma. Int J Rad Oncol Biol Phys. 2012;83(4):1252–1256. 10.1016/j.ijrobp.2011.09.046 PMC6324534 22280788

[14] Paech D , Dreher C, Regnery S, et al. Relaxation-compensated amide proton transfer (APT) MRI signal intensity is associated with survival and progression in high-grade glioma patients. Eur Radiol. 2019;29(9):4957–4967. 30809720 10.1007/s00330-019-06066-2

[15] Zukotynski KA , Fahey FH, Kocak M, et al. Evaluation of 18F-FDG PET and MRI associations in pediatric diffuse intrinsic brain stem glioma: a report from the pediatric brain tumor consortium. J Nucl Med. 2011;52(2):188–195. 21233173 10.2967/jnumed.110.081463 PMC3526809

[16] Zukotynski KA , Vajapeyam S, Fahey FH, et al. Correlation of 18F-FDG PET and MRI apparent diffusion coefficient histogram metrics with survival in diffuse intrinsic pontine glioma: a report from the pediatric brain tumor consortium. J Nucl Med. 2017;58(8):1264–1269. 28360212 10.2967/jnumed.116.185389 PMC5537615

[17] Löbel U , Hwang S, Edwards A, et al. Discrepant longitudinal volumetric and metabolic evolution of diffuse intrinsic pontine gliomas during treatment: implications for current response assessment strategies. Neuroradiology. 2016;58(10):1027–1034. 27438806 10.1007/s00234-016-1724-8 PMC5071138

[18] Tinkle CL , Duncan EC, Doubrovin M, et al. Evaluation of 11C-methionine PET and anatomic MRI associations in diffuse intrinsic pontine glioma. J Nucl Med. 2019;60(3):312–319. 30072503 10.2967/jnumed.118.212514 PMC6424234

[19] Lober RM , Cho YJ, Tang Y, et al. Diffusion-weighted MRI derived apparent diffusion coefficient identifies prognostically distinct subgroups of pediatric diffuse intrinsic pontine glioma. J Neurooncol. 2014;117(1):175–182. 24522717 10.1007/s11060-014-1375-8

[20] Gilligan LA , DeWire-Schottmiller MD, Fouladi M, DeBlank P, Leach JL. Tumor response assessment in diffuse intrinsic pontine glioma: comparison of semiautomated volumetric, semiautomated linear, and manual linear tumor measurement strategies. AJNR Am J Neuroradiol. 2020;41(5):866–873. 32354716 10.3174/ajnr.A6555 PMC7228153

[21] Sedlacik J , Winchell A, Kocak M, et al. MR imaging assessment of tumor perfusion and 3D segmented volume at baseline, during treatment, and at tumor progression in children with newly diagnosed diffuse intrinsic pontine glioma. AJNR Am J Neuroradiol. 2013;34(7):1450–1455. 23436052 10.3174/ajnr.A3421 PMC4249730

[22] Huang RY , Rahman R, Hamdan A, et al. Recurrent glioblastoma: volumetric assessment and stratification of patient survival with early posttreatment magnetic resonance imaging in patients treated with bevacizumab. Cancer. 2013;119(19):3479–3488. 23821555 10.1002/cncr.28210

[23] Buchlak QD , Esmaili N, Leveque JC, et al. Machine learning applications to neuroimaging for glioma detection and classification: an artificial intelligence augmented systematic review. J Clin Neurosci. 2021;89:177–198. 34119265 10.1016/j.jocn.2021.04.043

[24] Huang J , Shlobin NA, Lam SK, DeCuypere M. Artificial intelligence applications in pediatric brain tumor imaging: a systematic review. World Neurosurg. 2022;157:99–105. 34648981 10.1016/j.wneu.2021.10.068

[25] Prabhudesai S , Wang NC, Ahluwalia V, et al. Stratification by tumor grade groups in a holistic evaluation of machine learning for brain tumor segmentation. Front Neurosci. 2021;15:740353. 34690680 10.3389/fnins.2021.740353 PMC8526730

[26] Aboian M , Bousabarah K, Kazarian E, et al. Clinical implementation of artificial intelligence in neuroradiology with development of a novel workflow-efficient picture archiving and communication system-based automated brain tumor segmentation and radiomic feature extraction. Front Neurosci. 2022;16:860208. 36312024 10.3389/fnins.2022.860208 PMC9606757

[27] Peng S , Chen W, Sun J, Liu B. Multi-Scale 3D U-Nets: an approach to automatic segmentation of brain tumor. Int J Imaging Syst Technol. 2020;30(1):5–17.

[28] Liu Y , Stojadinovic S, Hrycushko B, et al. Automatic metastatic brain tumor segmentation for stereotactic radiosurgery applications. Phys Med Biol. 2016;61(24):8440–8461. 27845915 10.1088/0031-9155/61/24/8440

[29] Abdelaziz M , Cherfa Y, Cherfa A, Alim-Ferhat F. Automatic brain tumor segmentation for a computer-aided diagnosis system. Int J Imaging Syst Technol. 2021;31(4):2226–2236.

[30] Li G , Li L, Li Y, et al. An MRI radiomics approach to predict survival and tumour-infiltrating macrophages in gliomas. Brain. 2022;145(3):1151–1161. 35136934 10.1093/brain/awab340 PMC9050568

[31] Lazow MA , Nievelstein MT, Lane A, et al. Volumetric endpoints in diffuse intrinsic pontine glioma: comparison to cross-sectional measures and outcome correlations in the International DIPG/DMG Registry. Neuro-Oncology. 2022;24(9):1598–1608. 35148393 10.1093/neuonc/noac037 PMC9435485

[32] Emblem KE , Pinho MC, Zöllner FG, et al. A generic support vector machine model for preoperative glioma survival associations. Radiology. 2015;275(1):228–234. 25486589 10.1148/radiol.14140770

[33] Kazerooni AF , Khalili N, Liu X, et al. The Brain Tumor Segmentation (BraTS) Challenge 2023: *Focus on Pediatrics (CBTN-CONNECT-DIPGR-ASNR-MICCAI BraTS-PEDs)*. ArXiv [Preprint]. 2024 May 23:arXiv:2305.17033v7.

[34] Liu X , Bonner ER, Jiang Z, et al. From adult to pediatric: Deep learning-based automatic segmentation of rare pediatric brain tumors. In: Iftekharuddin KM, Chen W, eds. MedicalImaging 2023: Computer-Aided Diagnosis. San Diego, CA: SPIE; 2023:3. doi: 10.1117/12.2654245

[35] Drai M , Testud B, Brun G, et al. Borrowing strength from adults: transferability of AI algorithms for paediatric brain and tumour segmentation. Eur J Radiol. 2022;151:110291. 35405580 10.1016/j.ejrad.2022.110291

[36] Barkovich AJ , Krischer J, Kun LE, et al. Brain stem gliomas: a classification system based on magnetic resonance imaging. Pediatr Neurosurg. 1990;16(2):73–83. 2132928 10.1159/000120511

[37] Yushkevich PA , Piven J, Hazlett HC, et al. User-guided 3D active contour segmentation of anatomical structures: significantly improved efficiency and reliability. Neuroimage. 2006;31(3):1116–1128. 16545965 10.1016/j.neuroimage.2006.01.015

[38] Rohlfing T , Zahr NM, Sullivan EV, Pfefferbaum A. The SRI24 multichannel atlas of normal adult human brain structure. Hum Brain Mapp. 2010;31(5):798–819. 20017133 10.1002/hbm.20906 PMC2915788

[39] Thakur S , Doshi J, Pati S, et al. Brain extraction on MRI scans in presence of diffuse glioma: multi-institutional performance evaluation of deep learning methods and robust modality-agnostic training. Neuroimage. 2020;220:117081. 32603860 10.1016/j.neuroimage.2020.117081 PMC7597856

[40] Leao DJ , Craig PG, Godoy LF, Leite CC, Policeni B. Response assessment in neuro-oncology criteria for gliomas: practical approach using conventional and advanced techniques. AJNR Am J Neuroradiol. 2020;41(1):10–20. 31857322 10.3174/ajnr.A6358 PMC6975322

[41] Wen PY , van den Bent M, Youssef G, et al. RANO 2.0: update to the response assessment in neuro-oncology criteria for high- and low-grade gliomas in adults. J Clin Oncol. 2023;41(33):5187–5199. 37774317 10.1200/JCO.23.01059 PMC10860967

[42] Kline C , Jain P, Kilburn L, et al. Upfront biology-guided therapy in diffuse intrinsic pontine glioma: therapeutic, molecular, and biomarker outcomes from PNOC003. Clin Cancer Res. 2022;28(18):3965–3978. 35852795 10.1158/1078-0432.CCR-22-0803 PMC9475246

[43] Buczkowicz P , Hoeman C, Rakopoulos P, et al. Genomic analysis of diffuse intrinsic pontine gliomas identifies three molecular subgroups and recurrent activating ACVR1 mutations. Nat Genet. 2014;46(5):451–456. 24705254 10.1038/ng.2936 PMC3997489

[44] Vuong HG , Le HT, Ngo TNM, et al. H3K27M-mutant diffuse midline gliomas should be further molecularly stratified: an integrated analysis of 669 patients. J Neurooncol. 2021;155(3):225–234. 34796414 10.1007/s11060-021-03890-9

[45] Jansen MH , Veldhuijzen van Zanten SE, Sanchez Aliaga E, et al. Survival prediction model of children with diffuse intrinsic pontine glioma based on clinical and radiological criteria. Neuro Oncol. 2015;17(1):160–166. 24903904 10.1093/neuonc/nou104 PMC4483042

[46] Cai W , Steinberg SM, Bredella MA, et al. Volumetric MRI analysis of plexiform neurofibromas in neurofibromatosis type 1. Acad Radiol. 2018;25(2):144–152. 29097016 10.1016/j.acra.2017.09.004 PMC5794522

[47] Eichinger P , Alberts E, Delbridge C, et al. Diffusion tensor image features predict IDH genotype in newly diagnosed WHO grade II/III gliomas. Sci Rep. 2017;7(1):13396. 29042619 10.1038/s41598-017-13679-4 PMC5645407

[48] Chang P , Grinband J, Weinberg BD, et al. Deep-learning convolutional neural networks accurately classify genetic mutations in gliomas. AJNR Am J Neuroradiol. 2018;39(7):1201–1207. 29748206 10.3174/ajnr.A5667 PMC6880932

[49] Zhu X , Lazow MA, Schafer A, et al. A pilot radiogenomic study of DIPG reveals distinct subgroups with unique clinical trajectories and therapeutic targets. Acta Neuropathol Commun. 2021;9(1):14. 33431066 10.1186/s40478-020-01107-0 PMC7798248

