# Supplementary material for: Tumor volume features predict survival outcomes for patients diagnosed with diffuse intrinsic pontine glioma
Source: Neurooncol Adv. 2024 Aug 30;6(1):vdae151. doi: 10.1093/noajnl/vdae151 (PMC11492488; doi:10.1093/noajnl/vdae151)
Supplement: vdae151_suppl_Supplementary_Figues [file vdae151_suppl_supplementary_figues.docx]

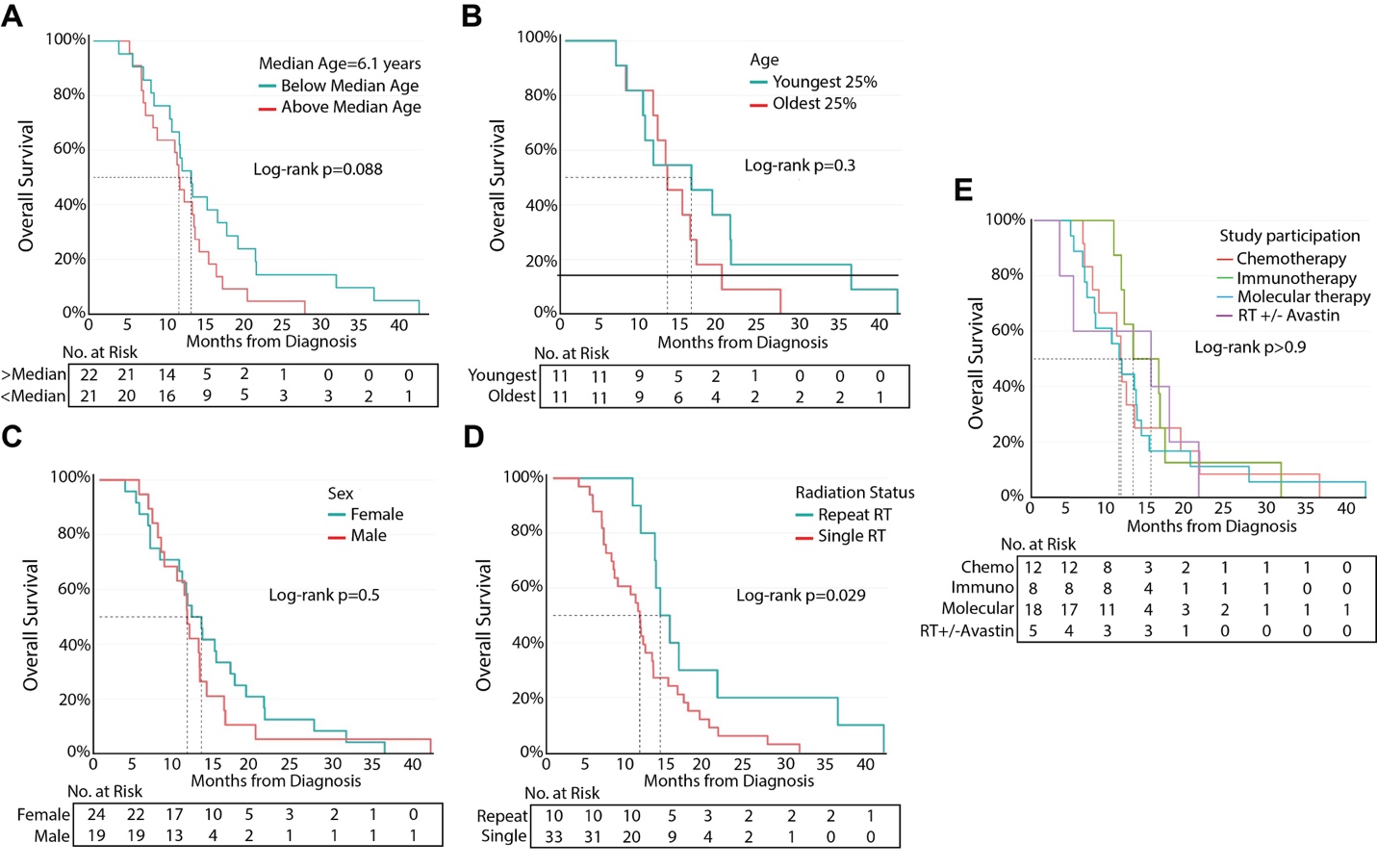


**Supplementary Figure 1. Kaplan-Meier plots for patient demographic and clinical features. (A-B)** Kaplan-Meier plots comparing patient overall survival outcomes based on patient age at diagnosis, **A:** youngest 50% (n=22) vs. oldest 50% (n=21); p=0.088, **B**: youngest 25% (n=11) vs. oldest 25% (n=11); p=0.3. **(C)** Kaplan-Meier plot comparing patient overall survival outcomes based on sex (female, n=24 vs. male, n=19); p=0.5, **(D)** single course (n=33) versus repeated courses of radiation therapy (n=10); p=0.029 and **(E)** treatment type including chemotherapy (n=12), immunotherapy (n=8), molecular therapy (n=18) or RT +/- Avastin (n=5); p=>0.9. All p-values based on Log-rank test.


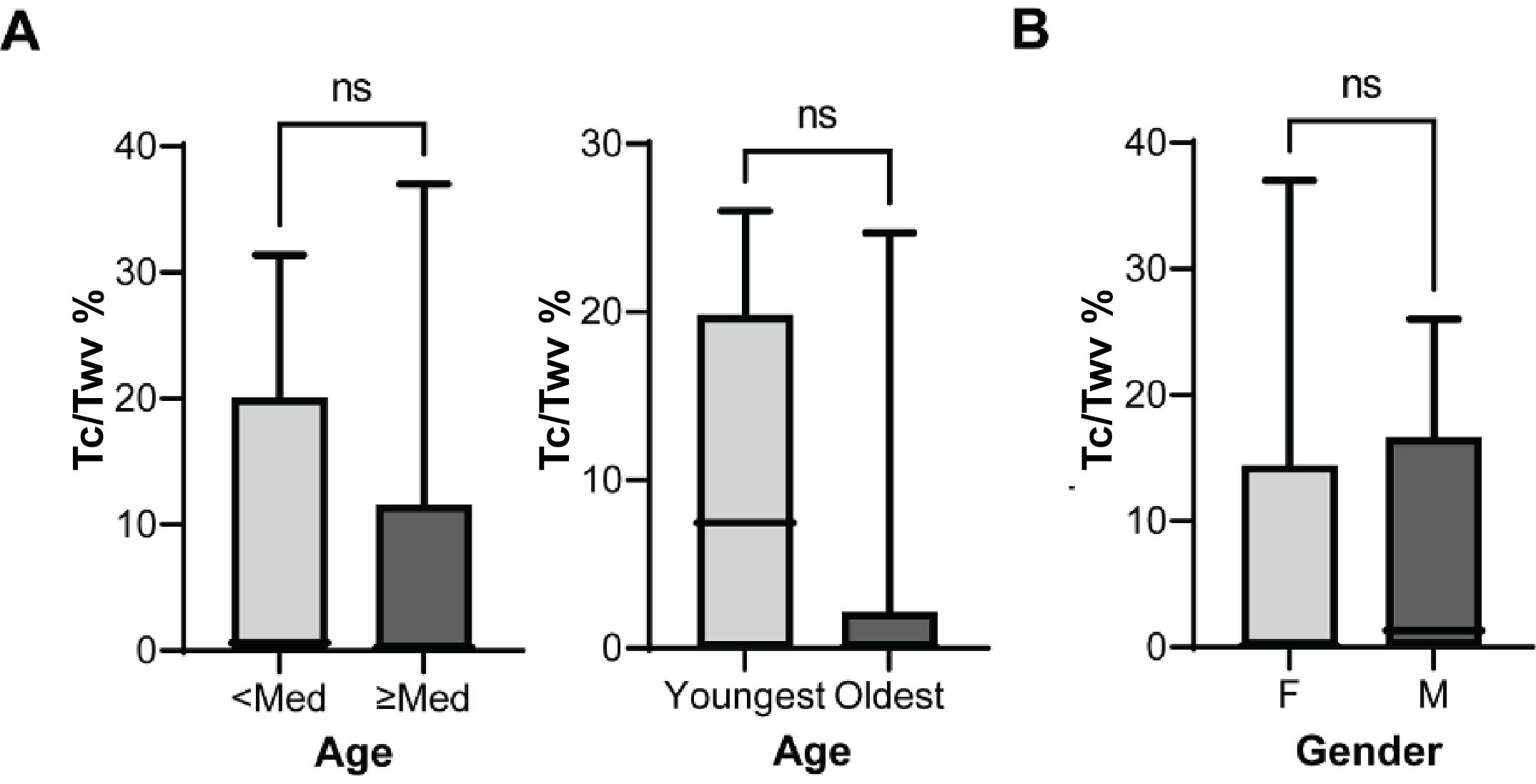


**Supplementary Figure 2. Effects of patient age and gender on diagnostic T_c_/T_wv_ ratio. (A) *Left:*** Diagnostic T_c_/T_wv_ ratio (%) between patients diagnosed below the median age of all patients (‘<Med’) verses those diagnosed at or above the median age of diagnosis (‘>=Med’). Median age =6.1 years. Mann-Whitney test, p=0.76. ***Right:*** Diagnostic T_c_/T_wv_ ratio (%) between the youngest patients (bottom 25%) and the oldest patients (top 25%). Mann-Whitney test, p=0.43. **(B)** Diagnostic T_c_/T_wv_ ratio (%) between female and male patients. Mann-Whitney test, p=0.48

**
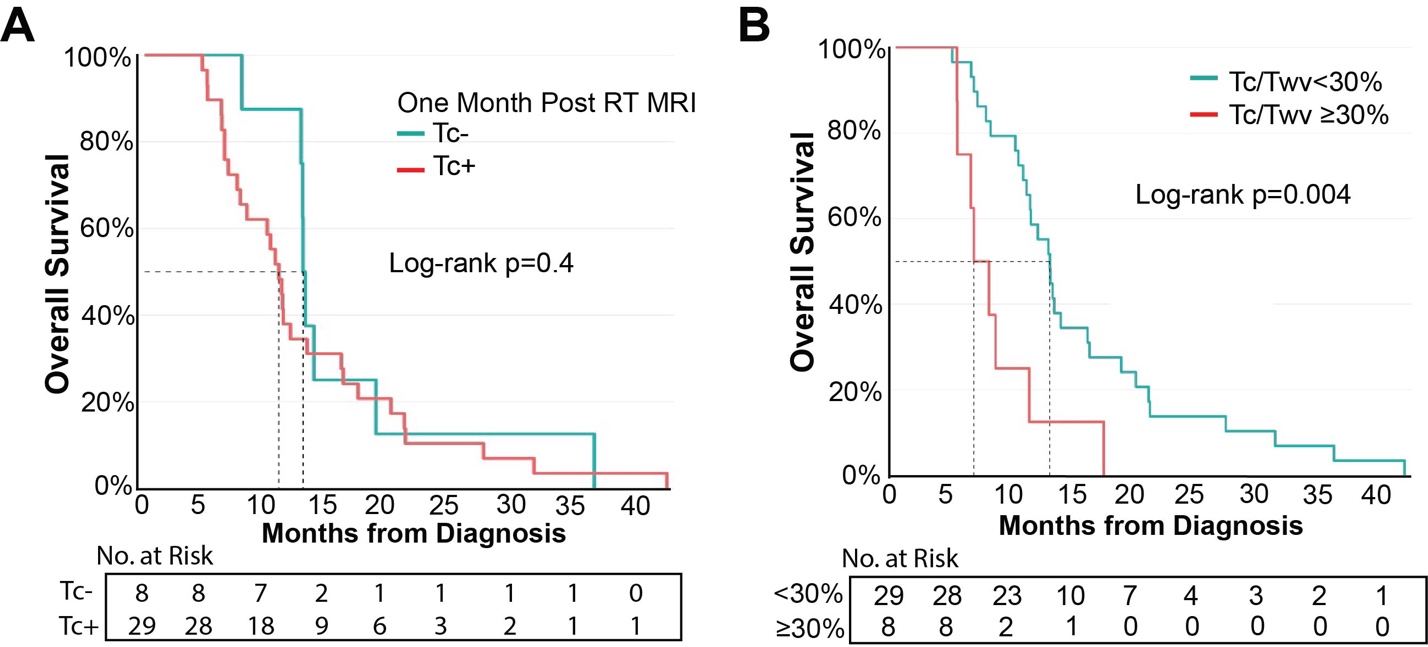
**

**Supplementary Figure 3. Kaplan-Meier plots representing the predictive value of contrast-enhancing tumor at <2 mos post radiation therapy on survival. (A)** Comparison of OS outcomes between patients based on the binary absence or presence of T_c_ at <2 mos post-RT (n=8 and n=29, respectively, Log-rank test, p=0.4). **(B)** OS outcomes for patients with ≥30% T_c_/T_wv_ ratio (n=8) at one-month post-RT compared to those with <30% (n=29). (Log-rank test, p=0.004).
