# Supplementary material for: Tumor volume features predict survival outcomes for patients diagnosed with diffuse intrinsic pontine glioma
Source: Neurooncol Adv. 2024 Aug 30;6(1):vdae151. doi: 10.1093/noajnl/vdae151 (PMC11492488; doi:10.1093/noajnl/vdae151)
Supplement: vdae151_suppl_Supplementary_Table [file vdae151_suppl_supplementary_table.docx]

| Publication Identifier | Volume MRI | Volume MRI <2 mos | Volume MRI 2-4 mos | Treatment during the <2 mos MRI | Treatment at the 2-4 mos MRI |
| --- | --- | --- | --- | --- | --- |
| 1 | FLAIR | FLAIR | FLAIR | Clinical Trial Chemotherapy | Clinical Trial Chemotherapy |
| 2 | T2 Propeller | NA | FLAIR | NA | Clinical Trial Chemotherapy |
| 3 | FLAIR | FLAIR | FLAIR | None | None |
| 4 | FLAIR | FLAIR | FLAIR | Clinical Trial Chemotherapy | Clinical Trial Chemotherapy |
| 5 | FLAIR | T2 | FLAIR | None | None |
| 6 | FLAIR+CON | FLAIR+CON | FLAIR+CON | None | None |
| 7 | FLAIR | FLAIR | FLAIR | Clinical Trial Chemotherapy | Clinical Trial Chemotherapy |
| 8 | FLAIR | FLAIR | FLAIR | Clinical Trial Chemotherapy | Clinical Trial Chemotherapy |
| 9 | FLAIR+CON | FLAIR+CON | FLAIR+CON | None | Avastin |
| 10 | FLAIR | FLAIR | FLAIR | Clinical Trial Chemotherapy | Clinical Trial Chemotherapy |
| 11 | FLAIR | FLAIR+CON | FLAIR | None | None |
| 12 | FLAIR | FLAIR | FLAIR | Clinical Trial Chemotherapy | Clinical Trial Chemotherapy |
| 13 | FLAIR | NA | NA | NA | NA |
| 14 | FLAIR | FLAIR+CON | FLAIR+CON | None | Clinical Trial Molecular Therapy + Chemotherapy |
| 15 | T2FSE | NA | T2 | NA | Clinical Trial Molecular Therapy |
| 16 | FLAIR | FLAIR | FLAIR | None | None |
| 17 | FLAIR | FLAIR+CON | FLAIR+CON | None | Clinical Trial Molecular Therapy + Chemotherapy |
| 18 | FLAIR | FLAIR+CON | FLAIR+CON | None | None |
| 19 | T2FSE | FLAIR+CON | FLAIR+CON | None | Clinical Trial Molecular Therapy + Chemotherapy |
| 20 | FLAIR+CON | FLAIR+CON | FLAIR+CON | None | Clinical Trial Molecular Therapy + Chemotherapy |
| Publication Identifier | Volume MRI | Volume MRI <2 mos | Volume MRI 2-4 mos | Treatment during the <2 mos MRI | Treatment at the 2-4 mos MRI |
| 21 | T2 | T2 | T2 | None | Chemotherapy |
| 22 | FLAIR | FLAIR+CON | NA | None | NA |
| 23 | FLAIR | NA | NA | NA | NA |
| 24 | T2FSE | FLAIR | FLAIR+CON | None | Clinical Trial Molecular Therapy + Chemotherapy |
| 25 | T2 | FLAIR+CON | FLAIR+CON | None | Clinical Trial Molecular Therapy + Chemotherapy |
| 26 | T2 | FLAIR+CON | T2 | None | None |
| 27 | FLAIR+CON | T2FSE | FLAIR | None | Clinical Trial Molecular Therapy + Chemotherapy |
| 28 | FLAIR+CON | FLAIR+CON | FLAIR+CON | None | None |
| 29 | T2FSE | FLAIR+CON | FLAIR | None | Clinical Trial Molecular therapy |
| 30 | FLAIR | FLAIR+CON | FLAIR+CON | None | Immunotherapy |
| 31 | T2FSE | DECEASED | DECEASED | DECEASED | DECEASED |
| 32 | FLAIR | FLAIR | FLAIR+CON | None | Immunotherapy |
| 33 | FLAIR+CON | FLAIR+CON | FLAIR+CON | None | None |
| 34 | FLAIR | T2 Propeller | T2 Propeller | None | Immunotherapy |
| 35 | FLAIR+CON | FLAIR+CON | FLAIR+CON | Immunotherapy | Avastin |
| 36 | FLAIR+CON | FLAIR+CON | FLAIR+CON | None | Immunotherapy |
| 37 | FLAIR | FLAIR+CON | FLAIR+CON | Clinical Trial Molecular therapy | Clinical Trial Molecular therapy |
| 38 | FLAIR | T2 | FLAIR | None | Immunotherapy |
| 39 | FLAIR+CON | FLAIR+CON | FLAIR+CON | Clinical Trial Molecular therapy | Clinical Trial Molecular therapy |
| 40 | FLAIR | FLAIR | FLAIR | Clinical Trial Chemotherapy | Clinical Trial Chemotherapy |
| 41 | FLAIR | FLAIR+CON | FLAIR+CON | None | None |
| 42 | T2 | T2 | NA | Clinical Trial Chemotherapy | Clinical Trial Chemotherapy |
| 43 | FLAIR+CON | NA | NA | NA | NA |
